# Supplementary material for: Moving the needle for oncology dose optimization: A call for action
Source: CPT Pharmacometrics Syst Pharmacol. 2024 May 22;13(6):909–18. doi: 10.1002/psp4.13157 (PMC11179700; doi:10.1002/psp4.13157)
Supplement: Supplementary file 1 — Data S1. [file PSP4-13-909-s001.docx]

**Supplementary Information:** **Survey Questions**

1. Are you primarily engaged in oncology R&D?
   1. Yes
   2. No
2. How valuable are pharmacodynamic biomarkers in the overall roadmap for dose optimization in oncology drug development?
   1. Indispensable
   2. Highly valuable
   3. Useful
   4. Limited value
   5. Not useful
3. Which of the following approaches are most relevant and valuable for selecting doses to bring forward in a dose optimization study?
   1. Translational modeling of antitumor activity (e.g., tumor growth inhibition) in preclinical models (e.g., patient-derived xenografts, syngeneic models, etc.)
   2. Quantitative Systems Pharmacology Models
   3. PK/PD modeling of pharmacodynamic biomarkers
   4. Clinical exposure-response modeling of tumor kinetics or other measures of antitumor activity in Phase 1/1b studies
4. Which of the following modalities poses the greatest challenge for dose optimization in oncology drug development?
   1. Next-generation cytotoxic agent
   2. Small molecule targeted agent
   3. Monoclonal antibody
   4. Multi-specific biologics (e.g., bi/tri-specific antibodies)
   5. Antibody-drug conjugates
   6. Cell therapies
5. Are strategies for dose optimization in other therapeutic areas/ indications relevant for oncology therapies?
   1. Yes
   2. No
6. Is randomized dose-ranging evaluation of efficacy and safety an obligate requirement for dose optimization in clinical development of oncology therapies?
   1. Yes
   2. No
